# Licensing Issues at Primary Clinics Resulting From the Omnibus Law in Indonesia: A Case Study From Surabaya City

**DOI:** 10.34172/ijhpm.9006

**Published:** 2025-11-05

**Authors:** Lilik Djuari, Samsriyaningsih Handayani, Sulistiawati Sulistiawati, Shafira Meidyana, Raudia Faridah Humaidy, Arya Ivan Mahendra, Ahmad Cholifa Fahruddin, Brahmaputra Marjadi

**Affiliations:** ^1^Department of Public Health and Preventive Medicine, Faculty of Medicine, Universitas Airlangga, Surabaya, Indonesia.; ^2^Community Medicine Coordination Bureau, Faculty of Medicine, Universitas Airlangga, Surabaya, Indonesia.; ^3^Faculty of Medicine, Universitas Airlangga, Surabaya, Indonesia.; ^4^School of Medicine, Western Sydney University, Campbelltown, NSW, Australia.

**Keywords:** Omnibus Law, Clinic Licensing, Regulation Shifting, Indonesia

## Abstract

**Background:**

Anecdotal evidence has indicated that Indonesian primary care clinics struggle to meet new licensing requirements under the new Omnibus Law in 2020. This study aimed to analyse the challenges associated with clinic licensing under this Law and identify potential practical solutions.

**Methods:**

This was a sequential exploratory mixed-method case study in Surabaya, the second-largest city in Indonesia. Participants were recruited using convenience sampling from a District Health Office WhatsApp group of 38% of primary clinics in Surabaya (85/223). We used a quantitative survey to assess primary clinics’ compliance with the new requirements. Subsequently, survey participants and key stakeholders were invited to two-phase focus group discussions (FGDs) where survey findings were reported and discussed. Quantitative data were analysed descriptively, and qualitative data were thematically analysed; both were synthesised at the final data analysis.

**Results:**

The survey response rate was 35% (30/85 clinics in the WhatsApp group). Compliance with the new regulation varied, with 17% of the clinics still awaiting license approval and 27% needing to obtain waste management permits. Eighteen survey participants and six key stakeholders participated in the FGDs. Five key challenges in primary clinic licensing were identified: regulation shifting, knowledge and perception, weakness in system design, time, and cost.

**Conclusion:**

The more complex licensing requirements have caused delays in license issuance for a convenience sample of primary clinics in this study. Ineffective communication and a lack of understanding between clinics, local government agencies, and external consultants have exacerbated these issues, leading to a heavy burden on clinic resources. To mitigate these challenges, policy-makers should prioritize simplifying sequential requirements, enhancing interagency coordination, and establishing clear communication channels—ensuring regulatory changes align with on-the-ground capacity and needs. By addressing these gaps, the licensing process can become more efficient and transparent, ultimately supporting clinic sustainability and healthcare access.

## Background

**Key Messages**

**Implications for policy makers**

- Streamline clinic licensing by creating a unified system with clear guidelines, timelines, and an online platform to reduce redundancy and confusion.
- Establish better communication channels, provide training on regulations, and create a liaison unit to resolve issues and rebuild trust between clinics and regulatory agencies.
- Implement strict oversight and accreditation for external consultants involved in the licensing process to prevent exploitation, ensure quality support, and rebuild trust between clinics and regulatory agencies.

**Implications for the public**

This case study highlights key barriers in primary clinic licensing under Indonesia’s updated regulations and offers practical solutions. By simplifying licensing processes, improving communication among stakeholders, and regulating external consultants, the proposed recommendations aim to reduce delays and operational challenges faced by clinics. For the public, these improvements mean better access to reliable primary healthcare services, as clinics can focus on delivering care rather than navigating complex administrative hurdles. Enhanced trust and coordination between clinics and regulatory bodies will promote more efficient healthcare systems, ensuring communities receive timely, high-quality care. Ultimately, these changes will strengthen Indonesia’s healthcare infrastructure, benefiting patients and fostering healthier communities.

Healthcare facilities are crucial in delivering services contributing to the nation’s health. In Indonesia, healthcare facilities are regulated under Law No. 17/2023, which mandates promotive, preventive, curative, and rehabilitative services coordinated by central and local governments and/or the public.^1^

 The Indonesian healthcare system categorises facilities into two tiers: first-tier facilities, including community health centres, primary clinics, independent medical practices, clinical laboratories, and blood transfusion units, and advanced-tier facilities, such as specialist clinics, hospitals, and pharmaceutical facilities.^1^ Among these facilities, anecdotal evidence through personal communications and observations indicated that primary clinics faced significant challenges in meeting national licensing requirements. This is especially concerning given the large numbers of primary clinics—14 564 nationwide in 2022, with 1732 in East Java, including 223 in Surabaya.^2,3^ According to the Surabaya City Health Office, 30% of these clinics did not fully comply with licensing standards.^4^ This non-compliance could disrupt their partnership with Health Social Security Service Agency (Badan Penyelenggara Jaminan Sosial or BPJS Health) network, affecting service availability to the community at large.^5^

 Since 2020, the licensing of primary clinics has been governed by multiple new health and non-health regulations, influenced by Law No. 11/2020 on Job Creation (Omnibus Law). The Law, which is primarily aimed at creating jobs for Indonesians, includes clauses about various types of business. The Law defined primary clinics as business entities and categorised them as high-risk under a Risk-Based Business Licensing system. Primary clinics are thus required to have a Business Identification Number and must adhere to strict standards verified by local governments. The Government Regulation No. 5/2021 and the Minister of Health Regulation No. 14/2021 outline these standards, emphasising compliance with non-health agencies, such as environmental authorities. In Surabaya, the local licensing processes are further detailed in the Mayor Regulation No. 41/2021, which specifies requirements, timelines, and applications through the Surabaya Single Window Alfa (SSW Alfa) system. Primary clinics must also obtain waste management permits from the City Environment Agency. These additional requirements were designed to improve primary clinics’ quality and ensure safer healthcare services for the public, particularly through enhanced safety standards in licensing and waste management. However, these regulations also pose significant administrative burdens and challenges for primary clinics, leading to delays in the licensing processes.

 This research aimed to analyse the challenges to primary clinic licensing under this regulatory suite and identify potential solutions for its application in Surabaya. To our knowledge, this study is the first in Indonesia to explore the challenges of primary clinic licensing under the Omnibus Law. By identifying key challenges and potential improvements, this study aims to provide insights for policy-makers in Surabaya and other regions facing similar regulatory challenges. This exploratory study in Surabaya is expected to serve as a case study for the rest of the country and other countries with similar challenges in regulating primary care services through legal instruments vis-à-vis meeting the mandate of broadening access to health for the population.

## Methods

 This research used an explanatory sequential mixed methods design.^6^ The study began with a quantitative survey followed by two-phase focus group discussions (FGDs) which were thematically analysed using a phenomenological approach.^7^ The qualitative findings were integrated to clarify the quantitative survey findings.

###  Quantitative Survey

 The quantitative survey was aimed at primary clinics in Surabaya using convenience sampling. Participants were recruited via a WhatsApp group coordinated by the District Health Office, representing 38% (85/223) of primary clinics in Surabaya. While this channel was practical and widely used among the targeted clinics, we acknowledge that it may not fully represent the broader population of primary clinics in Surabaya. All WhatsApp group members were invited to participate in the study. The inclusion criteria were primary clinics in Surabaya licensed under the Minister of Health Regulation 14/2021 who consented to the study. A reminder was broadcast one week after the initial invitation. Thirty clinics agreed to participate in the survey, representing 35% of WhatsApp group members (30/85) and 13% of licensed primary clinics in Surabaya (30/223). Each clinic was represented by its manager or another person in charge of the day-to-day running of the clinic.

 The survey was developed *de novo* as a self-assessment checklist based on Minister of Health Regulation 14/2021 requirements, including clinic characteristics, general requirements, specific requirements, facilities, rooms, equipment, structural organisation, human resources, and services. The survey wording was taken directly from the Regulation with Yes/No options to indicate each clinic’s self-reported compliance with full anonymity. Several open text boxes allowed participants to contextualise their responses. The survey draft was validated by a review by representatives from three primary clinics (one government-owned; one BPJS-linked privately owned; one non-BPJS privately owned) and one representative from the Indonesian Association of Clinics and Healthcare Facilities. The draft was accepted by the reviewers without any changes.

 Survey data were cleaned by using Statistical Package for the Social Sciences (SPSS) Frequencies command to identify erroneous entries, which were then marked as missing data. Missing data were excluded pairwise from analyses to preserve responses to other questions. Quantitative responses were analysed descriptively using IBM SPSS v. 25, and relevant open-text box responses were summarised. The purpose of this study was to explore the barriers faced by the clinics, rather than to examine associations or causal relationships. Given the exploratory nature and the limited sample in this study, our senior biostatistician advised that descriptive statistics were deemed appropriate for this investigation.

###  Qualitative Two-Phase Focus Group Discussions 

 Following the explanatory sequential mixed methods design,^6^ the quantitative data analysis results were presented and discussed in the subsequent qualitative arm of the study. The study’s qualitative component employed FGDs in two phases. Written consents were obtained from all FGD participants.

 The first FGD phase involved survey participants elaborating on their licensing experiences. An invitation embedded at the end of the survey yielded 18 FGD clinic-participants from the 30 clinics who participated in the survey (60%). The research team presented survey findings to these participants, who were divided into three groups: government-owned clinics (Group A, three participants), privately owned clinics in the BPJS Health network (Group B, 10 participants), and privately owned clinics outside the BPJS Health network (Group C, 5 participants). Each group discussion, guided by a facilitator and a note-taker, focused on interpreting survey findings and sharing clinic experiences. The researchers briefed all facilitators and note-takers about the study and trained them with a mock FGD practice.

 The FGD interview guide was based on the summary of survey findings. Participants were asked to first confirm and then elaborate the reasons for incomplete adherence to the Regulation. All questions were open-ended and practiced with facilitators and note-takers. The discussions were recorded, transcribed verbatim, and thematically analysed using a phenomenological approach.^7^ The FGD data were broken down to meaningful segments and labelled (open coding), which were then connected according to their relationships (axial coding), and refined to identify the main themes (selective coding).^8^ The open and axial coding processes were conducted independently by SM and RFH. Any coding discrepancies between the researchers were resolved through discussion until consensus was reached. The final selective coding process was performed by SM, RFH, and BM to establish the main themes. The themes were then arranged into a narrative form.^9^

 The second FGD phase engaged broader stakeholders identified from the survey and the first FGD results. Invitations were sent to three relevant agencies, followed by a reminder, with six officers participating to represent all three invited agencies. The second FGD began with presenting survey findings and results from the first FGD. Stakeholders from the Surabaya City Health Office (one participant), City Investment and One-Stop Services Office (two participants), and City Environment Agency (three participants) discussed key issues and proposed solutions. These discussions followed the same thematic analysis process as the first phase.

 Triangulation was employed by comparing participant statements across both FGD phases. The synthesised qualitative findings offered insights into, and potential solutions for, primary clinic licensing challenges as indicated in the survey results.

## Results

###  Quantitative Survey

 The survey response rate was 35% (30/65 clinics in the WhatsApp group), representing 13% (30/223) of all primary clinics in Surabaya, which introduced a potential risk of selection bias. Unresponsive clinics in the WhatsApp group, and clinics outside the WhatsApp group, may differ in terms of resources, engagement, or challenges faced. Therefore, the following findings should be interpreted as exploratory and context-bound, with limited generalisability.

 All primary clinics in this study reported to be supervised by a physician or a dentist. Most clinic-participants were within the BPJS Health network. Some clinics, especially recently established ones, still awaited health facility licenses (Table 1).

**Table 1:** Characteristics of the Primary Clinic

**Characteristics**		**n**	%*
Primary clinic	With dental services	25	83
	Without dental services	5	17
Supervisors	Physician	27	90
	Dentist	3	10
BPJS health network	Yes	19	63
	No	11	37
Health facility license	Yes	25	83
	On process	5	17
	No or expired	0	0
Ownership	Government	6	20
	Private	24	80
Establishment	<5 years	2	7
	5-10 years	6	20
	>10 years	22	73
Total		30	

Abbreviation: BPJS, Badan Penyelenggara Jaminan Sosial. * Denominators for percentages were the number of completed responses for each question.

 The compliance of these clinics with Minister of Health Regulation 14/2021 varied (Table 2). The unmet general requirements were interrelated, posing significant challenges. For instance, notarial decrees/acts for clinic establishment, which were a prerequisite for obtaining a Business Identification Number, were absent in 20% of clinics.

**Table 2:** Primary Clinic Compliance According to Ministry of Health Regulation 14/2021

	**n**	%*
**General requirements**		
Business identification number	24	80
Notarial decree/act for clinic establishment	27	90
Land certificate or lease agreement	30	100
Health facility permit	30	100
**Specific requirements**		
Healthcare waste management permit	30	100
Toxic and hazardous waste management agreement with an authorized party	30	100
Temporary storage sites of toxic and hazardous waste permit	22	73
Liquid waste management permit	22	73
**Facilities**		
Permanent and private building	30	100
Disability access	28	93
Smoke-free	29	97
Sign board (clinic type, name, and operational hours)	28	93
**Rooms**		
Administration		
Registration	30	100
Waiting room	27	90
Medical services		
Examination/consultation room	30	100
Dental services room	25	83
Medical treatment room	29	97
Emergency room	8	27
Additional medical services		
Radiology	0	0
Pharmacy	27	90
Laboratory	8	27
Sterilization room	27	90
Additional non-medical services		
Nursery room	29	97
Storage room	29	97
Toilets	30	100
Parking area	29	97
**Infrastructure**		
Ventilation	29	97
Lighting	30	100
Water and sanitation	30	100
Liquid waste management	25	83
Electricity	30	100
Medical gasses supply	7	23
Fire protection	29	97
Lightning protection system	13	43
Communication system	30	100
**Equipment**		
General examination tools	30	100
Single-use medical devices	30	100
Non-medical devices	30	100
Furniture	30	100
Documents and medical records	30	100
**Structural organizations**		
Supervised by a doctor or dentist	30	100
Supervisor is only in charge of one clinic	30	100
Supervisor has an active medical license	30	100
Have at least two physicians or dentists	30	100
**Human resources**		
Physicians	30	100
Dentists	25	83
Nurses	27	90
Midwiferies	18	60
Pharmacists	27	90
Nutritionists	1	3
All healthcare providers have active medical licenses	30	100
Appropriate workload distributions	24	80
Administrative personnel	24	80
Non-medical personnel	16	53
Security personnel	12	40
**Services**		
General medicine	30	100
Dental	25	83
Emergency	15	50
Referral	24	80
Homecare	17	57
Physical rehabilitation	0	0
Substance abuse rehabilitation	0	0
Pharmacy	29	97
Laboratory	14	47
Radiology	0	0
Nutrition	3	10
Medical devices sterilization	17	57

* Denominators for percentages were the number of completed responses for each question.

 Requirements related to facilities, rooms, equipment, and structural organizations were all met. Improvements were mainly needed for infrastructure, namely emergency room, laboratory, medical gasses supply, and lightning protection system. Human resources that were not widely available were midwives, nutritionists, non-medical personnel, security personnel, emergency, and homecare staff. Services that were often unavailable were radiology, physical rehabilitation, and substance abuse rehabilitation. All primary clinics already had their permit and agreements with an authorised party for toxic and hazardous waste management as prescribed. However, one-third of the clinics did not have temporary storage sites for toxic and hazardous waste, and they did not have liquid waste management permits.

###  Qualitative Focus Group Discussions

 The two-phase FGD identified several concerns under five themes: regulation shifting, knowledge and perception, weaknesses in system design,time, and cost.

###  Regulation Shifting

 The sudden implementation of new regulations created significant challenges for clinics and local government agencies. Clinics reported that the city-level technical guidelines were not well-adapted to their specific contexts, leading to difficulties in compliance. For instance, clinics that were part of a larger building faced structural constraints that conflicted with regulations: “*If I must demolish a wall [to meet space requirement], it’s not our clinic’s property—it belongs to the pharmacy [next door]*” (E, Private Clinic).

 Local agencies admitted they received no prior consultation or clear guidelines when the regulations were issued: “*But in my opinion, these agency people are the ones who make things difficult*” (G, Private Clinic). This lack of preparation led to miscommunication and inconsistent interpretation of the rules: “*Between agencies it is [sometimes] stated that liquid waste management is mandatory and [sometimes] not, well that’s it.*” (H, Government Clinic). Clinics expressed frustration over the limited dissemination of the new regulations and a desire for coordinated and consistent information from local government agencies. The fragmented coordination among agencies exacerbated the problem. While some agencies, such as the City Environment Agency, actively coordinated with others, others lacked clarity about their roles in the licensing process. Clinics called for more consistent and unified communication to address this gap.

###  Knowledge and Perception

 Disparities in knowledge and perceptions were prevalent among clinics and government agencies. Clinics, particularly those owned by non-health professionals, struggled to understand licensing requirements. Many relied on external consultants, whose advice often conflicted with the regulations, leading to confusion: “*If possible, we suggest not using an external consultant because the external consultant usually claims to be a consultant, [but] previously they were a service bureau. Because when we say A, later to the clinic it will be A min or A plus.*” (R, Surabaya City Health Office). Inconsistent guidance from local agencies, attributed to varying interpretations of regulations, further complicated the process. Clinics also highlighted the need for dedicated personnel to handle specific requirements, such as waste management, to ensure continuity and accountability, “…*once the person was changed, the story was different*…” (I, Government Clinic).

###  Weaknesses in System Design

 The Online Single Submission Risk-Based Approach (OSS-RBA) system posed significant challenges. Private clinics could access live chats and consultations through OSS, but government clinics were restricted to email communication, resulting in delayed responses: “*If I [consult through a phone] call, I can speak directly to the operator, that’s easy” *(N, Government Clinic). Many government clinics were unaware they needed a different platform (SSW Alfa) tailored to their requirements, contributing to confusion and inefficiency.

 At the city level, SSW Alfa clinics encountered unclear technical requirements and slow response times. Application errors were often revealed only after adverse decisions were issued, necessitating appeals and further consultations. Moreover, revisions were not communicated in their entirety, requiring clinics to attend multiple meetings to obtain advice on the various revisions they needed to undertake.

 Clinics in the BPJS Health network, mandated to use the OSS system, reported additional frustrations, including document loss and poor system maintenance. One participant noted, “*I was asked to wait for a month [for a compliance advice]*” (E, Government Clinic). Some participants observed that non-clinical businesses faced fewer issues with OSS, prompting clinics to prefer manual systems for greater flexibility in addressing minor deficiencies.

###  Time

 Due to lengthy processes, clinics found meeting licensing deadlines challenging. While licenses were theoretically valid for a limited period, the renewal process often exceeded a year, with some clinics experiencing delays of up to two years.

 Participants reported challenges in repeatedly visiting local agencies: *“... [we] visited again [and] still wrong again...” *(J, Private Clinic). This was the reason why some clinics resorted to employing external consultants to expedite the process. However, even with these measures, clinics faced delays, especially for licenses requiring extensions to maintain BPJS Health partnerships: “*I have been taking care of it for one and a half years, almost two years, but the license has not been issued”* (S, Private Clinic). Different licensing purposes under the same agency also had variable processing times, further complicating the timeline for completion.

###  Cost

 The regulatory shift significantly increased licensing costs. Although the licensing process was officially free, associated requirements, such as waste management and water testing, imposed substantial financial burdens: “*To meet the requirement for a waste management permit, we spent sixty-one million rupiah”* (E, Private Clinic).

 Costs varied depending on the clinic’s specific circumstances and the use of external consultants, whose fees were often arbitrary and expensive. Some consultants misrepresented their credentials, leading to misinformation and further clinic expenses: “W*hen asked for the name, the name mentioned was non-existent in our department, so that person was not related to the agency at all*…” (R, Government Agency). Government agencies advised clinics to avoid external consultants to reduce costs and rely instead on agency-provided consultations, free of charge: “S*o, why is it expensive? It’s because of these consultants. They set their fees as they wish*…” (U, Government Agency).

 Water testing, in particular, was noted as a costly and time-consuming requirement. Clinics opting for private waste management providers faced higher fees later from the related government agency, which involved long wait times and repeated monthly testing. Participants emphasised the need for more affordable and efficient testing processes.

###  Synthesised Findings

 In summary, the quantitative and qualitative findings pointed to complex and interrelated systemic and practical issues in primary clinic licensing which created barriers to compliance. The qualitative data shed light on the low compliance found in the survey. Underlying issues included a combination of inconsistent translations of national policies and regulations into the local city context, lack of coordination among various agencies (some of whom were previously not involved in primary clinic licensing), and high burdens on clinics due to inefficient systems, unclear fee structures, and questionable consultants.

## Discussion

 The implementation of Minister of Health Regulation 14/2021, in line with the Omnibus Law on Job Creation (Law 11/2020), has significantly impacted primary clinic licensing procedures by introducing new requirements for healthcare facility permits, including tightening environmental permits. This study found that these changes have caused delays in issuing health facility licenses for many clinics, threatening their survival and access to primary care for their local communities. The Community Health Governance theory^10^ highlights the challenges of implementing health policies in decentralized systems, where central governments design policies but rely on local authorities for execution to improve community health outcomes. However, implementation gaps often arise due to local governments’ limited resources, weak accountability mechanisms, and insufficient motivation to enforce regulations effectively.^10^ These theoretical tensions resonate with the empirical realities observed in this study. While the regulation aims to streamline healthcare licensing, which indeed will improve health service quality and patient safety, its decentralized implementation has paradoxically prolonged licensing delays, jeopardising clinic operations and community access to primary care.

 While most clinics in this study met the necessary facility requirements, several clinics still waited for their license mainly due to their inability to meet general and specific requirements. The interdependency of these requirements exacerbated the delays, as some prerequisites must be completed before others can proceed. The survey findings in this study identified the critical aspects which were lacking among some clinics, which could hinder their licensing attainment. The deficiencies in infrastructure, human resources and services provided by the clinics are interconnected, which may complicate their resolution. However, there are alternatives that may be considered by the clinics, at least as temporary measures. For example, clinics that collaborate with BPJS Health (the Health Social Security Service Agency) may enter into a collaboration agreement with private laboratories and private midwives rather than providing their own laboratory and midwifery facilities and staff.^11^ The lack of a dedicated Emergency Room may be temporarily resolved by providing emergency services and medications in the general treatment room.^12^

 Issues with primary clinic licensing in Indonesia existed even before the new regulation, with bureaucratic complexity and prolonged licensing durations being significant problems.^13-15^ Similarly, a study in Ukraine highlighted challenges in obtaining licenses for private healthcare facilities, primarily due to bureaucratic procedures regulated by the Minister of Health with contradictory requirements and inconsistencies that complicate compliance and operational planning. There is also a threat of license revocation without clear justification, which poses a risk to investment and stability.^16^ Similar licensing challenges were observed in Malaysia, where clinic licensing procedures also follow a two-stage process (establishment approval followed by operational licensing), mirroring the Indonesian system. A study in Malaysia^17^ identified specific regulatory gaps, particularly in air ventilation system compliance, whereas our study highlights obstacles in temporary storage of toxic and hazardous waste permits and liquid waste management permit—both of which impose additional renovation costs and financial burdens on applicants under the new regulation, as well as emotional stress due to prolonged uncertainty. Notably, regulatory misalignment between authorities and applicants persists in Malaysia,^17^ akin to our findings. Despite support from the central Ministry of Health, local regulators face capacity constraints in conducting field verifications, leading to inefficiencies and delays.^17^

 Although the environmentally related regulations under the Indonesian Omnibus Law aimed to align with World Health Organization (WHO) recommendations for healthcare waste management to consider the impact of healthcare activities on the environment,^18,19^ a lack of regulatory understanding and miscommunication between agencies and clinics has created obstacles in securing primary clinic licenses for participants in this study. The climate change issue has become a worldwide agenda and similar concerns regarding the burden of implementing climate change mitigation for primary care services have been raised in other countries, including Germany and Australia.^18,19^ In these developed countries, barriers to implementing climate change mitigation measures also came from a lack of knowledge and understanding about the protocol and unclear practical guidance for implementing these measures.^18,19^ In addition, our study found that the involvement of external consultants with questionable credentials, who exploited the circumstances to impose additional economic burdens on the clinics, further complicated the situation and created mistrust between the clinics and the regulatory agencies.

 The primary clinics and their licensing stakeholder agencies in this study acknowledged the significant impact of these Indonesian regulatory changes on licensing. The abrupt implementation led to insufficient understanding among primary clinics and agencies about the new regulations, causing communication and coordination issues. These impacts reflected a volatility, uncertainty, complexity, and ambiguity (VUCA) environment.^20^ The VUCA environment poses a significant threat to clinics as primary care providers by creating conditions that make decision-making, patient care, and overall healthcare delivery more challenging.^20^ Volatility, characterised by rapid and unpredictable changes, forces clinics to constantly adjust to fluctuating healthcare demands and, as this study revealed, the regulatory shifts. This constant state of change can overwhelm primary care providers, resulting in inconsistent care and a reduced ability to forecast patient needs or resource allocation. Uncertainty around the clinic license issuance in this study further exacerbates the situation by hindering the ability to predict future trends or needs in patient care. Due to the unpredictable nature of healthcare demands or evolving regulations, clinics may struggle with planning for staffing, patient services, or adopting new technologies. Without a clear understanding of future challenges, clinics may be ill-prepared, decreasing service quality and patient satisfaction. Complexity emerges from the detailed new regulations requiring significant adjustments in both physical infrastructure and clinic governance systems, including costly modifications to meet environmental and waste management standards. Many clinics found themselves unprepared for these comprehensive changes that affected nearly all aspects of their operations. Ambiguity has been particularly pronounced due to conflicting information from external consultants, whose interpretations often diverged from official local authority guidelines. This ambiguity created confusion about compliance requirements and eroded trust in the licensing process. Addressing these challenges through vision, understanding, clarity, and agility can enhance the healthcare system’s resilience.^20^

 This study indicated that confusion around the new licensing requirements may negatively impact on the trust between the clinics and the government. This mistrust can pose significant threats to public well-being by undermining the effectiveness of healthcare delivery. The mistrust against the government may also trickle down to the public if long-existing clinics which have served their local communities are forced to pause their services while completing their licensing requirements, or cease practicing altogether. When citizens lack trust in their government, they may be less likely to engage with health services, leading to delayed or missed care and exacerbating health problems.^21^ Furthermore, if individuals perceive the healthcare system as ineffective, unfair, or financially burdensome (eg, due to a broken partnership with BPJS), their reluctance to seek care increases, leaving them vulnerable to untreated health issues. This mistrust also hampers the government’s ability to implement and sustain health policies, as public cooperation is essential for the success of health programs. In countries with low trust, citizens may also resist or disengage from health initiatives, including preventive measures or vaccination campaigns, thus perpetuating cycles of poor health outcomes.^21^ The cumulative result is a weakened healthcare system, with higher rates of preventable diseases, increased healthcare costs, and a population less likely to support necessary reforms or public health measures, creating a dangerous feedback loop that threatens public health and safety.

 This complex regulatory environment in Indonesia, typified by its second-largest city, Surabaya, underscores the need for better communication and coordination among all stakeholders involved. The VUCA environment highlights the importance of comprehensive communication and guidance to ensure all parties are adequately prepared to navigate the new requirements efficiently. Addressing these challenges is crucial for streamlining and improving the primary clinic licensing process and rebuilding trust among clinics, regulatory bodies, and other stakeholders.

 Several factors limited this study. Firstly, the study’s participants only represented 13% of all primary clinics in Surabaya (30/223). We acknowledge the risk of selection bias and the limited generalisation of our findings. The lack of official sampling frame at the City Health Office led to our use of their WhatsApp group as the only available alternative. A larger, preferably national study with a better sampling frame is needed to confirm the findings of this case study. Secondly, the relatively high level of compliance among study participants may have been due to self-selection bias, where non-compliant clinics were reluctant to participate in both arms of the study. Despite this potential bias, participants still reported high levels of challenges and frustration, indicating that even higher levels may exist among primary care clinics with lower compliance. Thirdly, the key stakeholders who participated in this study were mainly the top executives and may not have fully voiced the perspectives of their frontline staff who engage directly with primary clinics. However, they reported having been contacted directly by some clinics to troubleshoot their challenges. Fourthly, the choice of Surabaya as the study setting was convenient based on the research team’s familiarity with the city. Due to provincial and district/city differences across Indonesia, different sets of challenges may exist in other areas. Further research is needed to identify other challenges and their contributing factors, including how some clinics may have complied better to the licensing requirements than others.

 The findings point toward critical systemic improvements needed to transform regulatory challenges into opportunities for healthcare strengthening. A harmonized digital licensing platform with transparent procedures and standardized timelines could reduce bureaucratic redundancies while maintaining oversight rigor, particularly when complemented by dedicated liaison units and structured training programs to align stakeholder understanding. Such reforms would address the current knowledge gaps and communication breakdowns while preventing exploitation through accredited third-party consultant systems. Beyond institutional benefits, these measures promise cascading impacts on healthcare accessibility—enabling clinics to reallocate resources from administrative burdens to service delivery, thereby enhancing both care quality and community trust in the decentralized health system. When regulatory processes become more dependable and collaborative, the entire healthcare ecosystem stands to gain, including clinics operate with greater stability, local agencies execute oversight more effectively, and communities ultimately receive more reliable primary care services.

## Conclusions

 This study highlights significant licensing challenges for a convenience sample of primary clinics in the second-largest city in Indonesia under a new suite of national and local regulations. The new requirements have introduced complexities for health facilities licensing, leading to delays in license issuance from interdependent requirements that must be obtained in a specific sequence. Communication breakdowns and a lack of understanding between clinics, local government agencies, and external consultants exacerbated these issues, leading to a heavy burden on clinic resources. The study underscores the need for improved communication, stakeholder engagement, and coordination to navigate the regulatory changes effectively, such as establishing standardized digital platforms for real-time policy updates, interagency training workshops to align regulatory interpretations, and dedicated government liaisons to provide authoritative guidance. By addressing these challenges, the licensing process can be streamlined, fostering a more efficient and transparent environment for clinics.

## Acknowledgements

 We sincerely thank the clinic representatives, Surabaya City Health Office, City Investment and One-Stop Services Office, and Environment Agency for their invaluable insights, data, and support, which were essential to this research.

## Disclosure of artificial intelligence (AI) use

 The authors used Grammarly software while writing the manuscript to assist in identifying and correcting grammatical mistakes.

## Disclaimer

 This report reflects the authors’ findings based on data from Surabaya and may not fully represent conditions elsewhere in Indonesia. While every effort was made to ensure accuracy, the results should be interpreted considering the study’s limitations. Recommendations are for informational purposes and should be adapted to local contexts.

## Ethical issues

 This study was approved by the Health Research Ethics Committee, Faculty of Medicine, Universitas Airlangga (81/EC/KEPK/FKUA/2022 with amendment 35/EC/KEPK/FKUA/2025). FGD participants were given a Clinic Pocket Book on the Minister of Health Regulation 14/2021 (ISBN: 978-623-99961-0-9) issued by the Department of Public Health and Preventive Medicine, Faculty of Medicine, Universitas Airlangga.

## Conflicts of interest

 Lilik Djuari works in a primary clinic. Other authors declare that they have no conflicts of interest.
